# Dense Oil in Water Emulsions using Vortex-Based Hydrodynamic Cavitation: Effective Viscosity, Sauter Mean Diameter, and Droplet Size Distribution

**DOI:** 10.1021/acs.iecr.3c04555

**Published:** 2024-03-11

**Authors:** Mukesh Upadhyay, Akshay Ravi, Vivek V. Ranade

**Affiliations:** Multiphase Reactors and Intensification Group Bernal Institute, University of Limerick, Limerick V94T9PX, Ireland

## Abstract

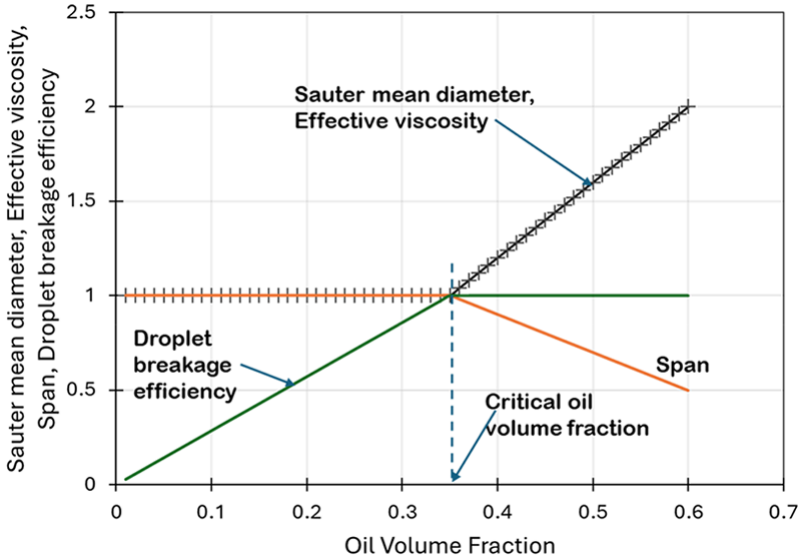

Vortex-based hydrodynamic cavitation offers an effective platform for producing emulsions. In this work, we have investigated characteristics of dense oil in water emulsions with oil volume fractions up to 60% produced using a vortex-based cavitation device. Emulsions were prepared using rapeseed oil with oil volume fractions of 0.15, 0.3, 0.45, and 0.6. For each of these volume fractions, the pressure drop as a function of the flow rate of emulsions through the cavitation device was measured. These data were used for estimating the effective viscosity of the emulsions. The droplet size distribution of the emulsions was measured using the laser diffraction technique. The influence of the number of passes through the cavitation device on droplet size distributions and the Sauter mean diameter was quantified. It was found that the Sauter mean diameter (*d*_32_) decreases with an increase in the number of passes as *n*^–0.2^. The Sauter mean diameter was found to be almost independent of oil volume fraction (α_o_) up to a certain critical volume fraction (α_oc_). Beyond α_oc_, *d*_32_ was found to be linearly proportional to a further increase in oil volume fraction. As expected, the turbidity of the produced emulsions was found to be linearly proportional to the oil volume fraction. The slope of turbidity versus oil volume fraction can be used to estimate the Sauter mean diameter. A suitable correlation was developed to relate turbidity, volume fraction, and Sauter mean diameter. The droplet breakage efficiency of the vortex-based cavitation device for dense oil in water emulsions was quantified and reported. The breakage efficiency was found to increase linearly with an increase in oil volume fraction up to α_oc_ and then plateau with a further increase in the oil volume fraction. The breakage efficiency was found to decrease with an increase in energy consumption per unit mass (*E*) as *E*^–0.8^. The presented results demonstrate the effectiveness of a vortex-based cavitation device for producing dense oil in water emulsions and will be useful for extending its applications to other dense emulsions.

## 1 Introduction

Emulsions play pivotal in many industries across diverse sectors such as food processing (milk products, ice creams, and salad dressings)^1−3^ healthcare (drugs and active pharmaceutical ingredients)^4−6^ personal care (cosmetics, fragrances, and beauty care)^7^ and other industries.^8^ Single emulsions are simplest form of emulsions categorized in two major types as oil in water (O/W) and water in oil (W/O).^9^ Numerous emulsion preparation methods and equipment are available, ranging from high-pressure homogenizers, microfluidization, and rotor-stator systems to ultrasonication, membranes, and more.^10^ These methods are classified based on energy input into high-energy methods (e.g., high-pressure homogenization, colloid mills, ultrasonication, and microfluidization) and low-energy methods (e.g., emulsion inversion point and phase inversion temperature).^11^ In general, significant energy input is required for droplet generation, and energy input increases significantly for realizing smaller droplet sizes.^9^

Several new technologies like acoustic and hydrodynamic cavitation, irradiation, high-hydrostatic pressure, microwave, pulsed electric field, and ohmic heating are emerging for emulsions.^12^ Hydrodynamic cavitation (HC) is one of the most promising technologies for producing emulsions.^13^ HC is a process of the generation, growth, and collapse of vapor cavities in liquids. HC is achieved by realizing low pressure (approaching the vapor pressure of a liquid at the operating temperature) zones where cavities are generated. When these cavities travel to a region of higher pressure, they implode (collapse) and generate intense shear and localized hot spots.^14^ This intense shear can be harnessed to produce fine emulsions. Ramisetty et al.^15^ use a venturi-based HC device to generate a coconut oil in water emulsion. They studied parameters like inlet pressure and passes through the cavitating zone to control the droplet size distribution. Zhang et al.^16^ used a circular venturi-based HC device to intensify the emulsion process for chitosan nanoparticle synthesis. Parthasarathy et al.^17^ utilized a liquid whistle HC reactor (LWHCR) for generating palm oil-based sub-micrometer emulsions. Carpenter et al.^18^ reported that the energy density required for acoustic cavitation (∼10^7^ kJ/m^3^) is much higher than that for HC (∼10^6^ kJ/m^3^) for the processing of emulsions. Thus, HC technology is a much more energy efficient technique than acoustic cavitation and a suitable choice for dense emulsions.

One of the first works on a utilizing vortex-based HC device for producing liquid–liquid emulsions was reported by Thaker and Ranade.^19^ Thaker and Ranade^20^ performed extensive experiments to investigate the influence of various parameters on the droplet size distribution (DSD) to produce an oil in water emulsion with an oil volume fraction (α_*o*_) up to 0.15. In this work, we have investigated the characteristics of emulsions with high oil volume fractions up to 0.6. We also investigated the influence of the oil volume fraction on effective viscosity. Turbidity and absorbance measurements were used to estimate the Sauter mean diameter. The new data on DSD, effective viscosity, Sauter mean diameter, span, and droplet breakage efficiency and new correlations valid for oil volume fractions up to 0.6 are presented.

DSD is a critical quality attribute (CQA) intrinsically related to rheology,^21^ appearance,^22^ and overall emulsion quality. It is influenced by several parameters, including the homogenization technique,^23,24^ the physical properties of the liquid phase,^25^ the type of emulsifier used,^26,27^ and the temperature protocol.^28^ Numerous studies in the literature have delved into the influence of DSD on viscosity. Specifically, researchers have found that polydispersity (a wide DSD) in emulsions leads to a relatively small increase in viscosity when compared to emulsions with a monodisperse DSD.^29−31^ Additionally, it is noteworthy that combining two or more distinct droplet size groups yields result in a dispersion with lower viscosity than one consisting of a single particle size group.^32−34^ In recent study, Mugabi and Jeong^35^ investigated the influence of DSD (polydispersity) on the emulsion viscosity by adjusting the polydispersity of an emulsion through precisely mixing monodispersed emulsions with different droplet sizes. Interestingly, their findings show modest changes in viscosity with regard to DSD. Several models have been developed for estimating the viscosity of emulsions. For dilute emulsions, viscosity is estimated using Einstein’s well-known equation.^36^ In the case of dilute emulsions, droplets have a relatively small effect on the emulsion viscosity. For higher oil/dispersed phase volume fractions, the crowding of droplets results in higher hydrodynamic interactions, ultimately altering the viscosity of the system.^31^ Allouche et al.^37^ monitored the conductivity and viscosity during emulsion phase inversion. They measured viscosity by detecting the torque produced by the relative motion of the U-type anchor impeller with respect to the vessel’s content using the RFS II rheometer. Urdahl et al.^38^ determined the effective viscosity based on torque and rotational measurements of a high-pressure loop wheel filled with the desired fluid. A similar wheel flow simulator was employed to measure effective viscosity in water in oil emulsions under various temperature and pressure conditions, up to 100 bar.^39^ In this work, the interest is in estimating effective viscosity rather than fuller rheological characterization of emulsions. We estimated the effective viscosity of emulsions by measuring flow versus pressure drop data. These data and the pressure drop correlation developed for Newtonian fluids were used to estimate effective viscosity of emulsions.

Numerous advanced techniques (laser diffraction, dynamic light scattering, and multiangle static light scattering) are available for accurate measurement of the size distribution of emulsions/dispersions.^40−42^ In this work, we have used a laser diffraction technique to measure the DSD of oil in water emulsions. The influence of the number of passes through the vortex-based cavitation device and the volume fraction of oil on DSD is investigated. The key characteristic parameters of DSD such as the Sauter mean diameter and span are reported. The fully resolved measurements of DSD using laser diffraction are time-consuming. Therefore, alternative methods based on turbidity or UV (ultraviolet) absorbance measurements were investigated.^43^ There are numerous reports on the use of turbidimetric techniques for determining either an average size or distribution in polydispersed suspensions such as polylatexes^44^ and oil in water emulsions.^45^ Multiwavelength UV spectroscopy measurements were used for characterization of polymer and copolymer latex emulsions.^46^ In a recent study conducted by Aspiazu et al.,^47^ they used a wavelength exponent method to assess changes in turbidity across various wavelengths. In this work, we used a commercial turbidity meter as well as light absorbance as a function of the volume fraction of oil in the emulsion. The data were used to estimate characteristic droplet diameter, which was then used to estimate the Sauter mean diameter (*d*_32_) of the emulsions.

The measured data on DSD and the Sauter mean diameter were used to calculate droplet breakage efficiency. The influence of the number of passes or, in other words, energy consumption per unit weight of emulsion, and the oil volume fraction on droplet breakage efficiency was quantified and discussed. The presented results will provide a sound basis and experimental data for extending applications of vortex-based hydrodynamic cavitation to emulsions and related areas.

## 2 Experimental Section

**Figure 1:**
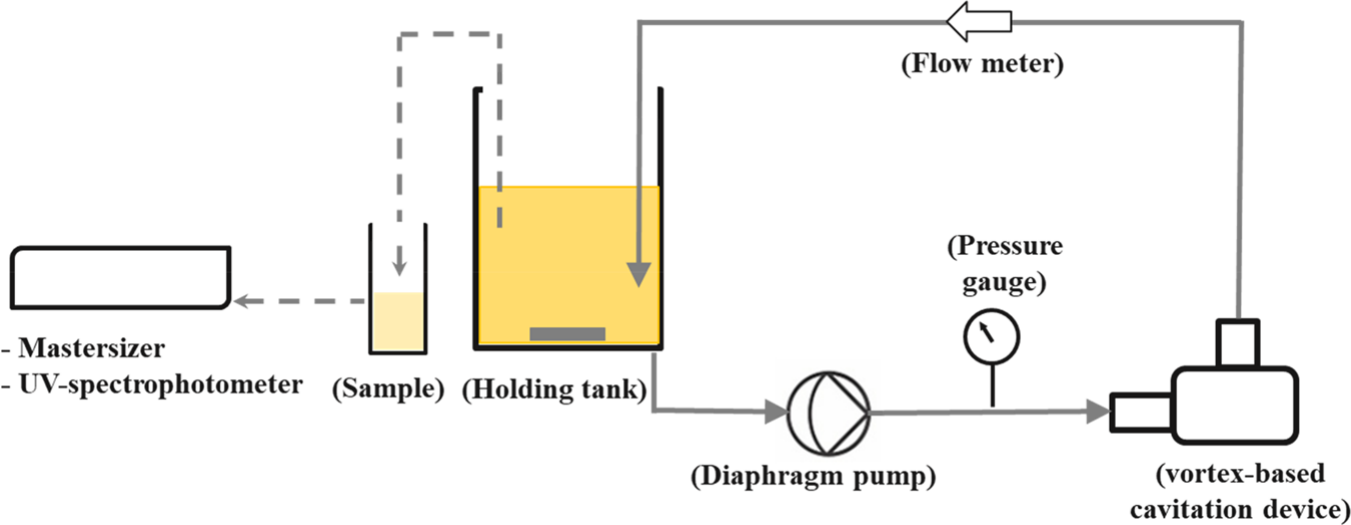
Schematic of the experimental setup.

The oil in water emulsions were produced using vortex-based HC using the experimental setup shown schematically in Figure 1. The throat diameter of the diode (*d*_T_) was 3 mm. The rest of the dimensions of diodes with reference to the throat diameter were the same as those reported in previous work.^48^ The experimental procedure consists of the following steps. First, the continuous phase water was prepared by adding 2% (w/v) TWEEN 20 (MP Biomedicals, LLC, France) surfactant. The monolayer coverage for the highest oil volume fraction of oil considered in this work (0.60) is less than 0.1% (by weight), assuming the Sauter mean diameter of emulsion as 1 μm. Based on our previous experience, we added significantly excess surfactant (2%) over the amount required for monolayer coverage for all our experiments to ensure the effective prevention of droplet coalescence on the measured DSD.^49^ After the surfactant was dissolved, rapeseed oil (Newgrange Gold, Tesco Ireland) was added in the desired quantity to achieve the set volume fraction of oil. The experiments were performed with four different rapeseed oil volume fractions, i.e., 0.15, 0.30, 0.45, and 0.60. The experiment was performed with a total volume of 500 mL. The contents of the holding tank were mixed using a magnetic stirrer operated at 300 rpm for 10 min. The coarse emulsion created by magnetic stirring was pumped through the vortex-based cavitation unit using a diaphragm pump (Sinleader, Model SL-DP-16). A pressure gauge (EN 837-1, WIKA) with a pressure range of 0–250 kPa (accuracy of ±2.5% full scale) was used for pressure drop measurements. The pressure drop readings were taken by averaging over at least one min, and these exhibited good reproducibility with a rather small standard deviation. A precalibrated digital mass flow meter (Us211M) was used for monitoring of flow rate. For investigating the influence of oil volume fraction on the effective viscosity of the emulsions, the emulsion produced after 100 passes through the HC device was used to record flow rate versus pressure drop data. All measurements were carried out three times. The material properties and experimental conditions are listed in Table 1. The Reynolds number mentioned in Table 1 is based on the viscosity of water, since the effective viscosity of the emulsions was not known a priori. The definition of cavitation number for the vortex-based cavitation devices is not straightforward. Ranade et al.^14^ suggested that the cavitation number for vortex-based cavitation devices may be calculated based on the maximum tangential velocity in the vortex chamber. Recently, Gode et al.^56^ proposed a correlation between pressure drop across the cavitation device and maximum tangential velocity in the vortex chamber. Based on that, the cavitation number , where *P*_2_ is the downstream pressure, *P*_v_ is the vapor pressure of water at the operating temperature, Δ*P* is the pressure drop across the HC device, and *Ca* is the cavitation number, for the experiments conducted in this work was nearly one.

**Table 1:** System Geometry, Material Properties, and Operation

description	value
throat diameter of HC device, *d*_*T*_ (m)	0.003
rapeseed oil density, ρ_o_ (kg/m^3^)	915
rapeseed oil viscosity, μ_o_ (mPa·s)	62
water density, ρ_w_ (kg/m^3^)	997
water viscosity, μ_w_ (mPa·s)	0.7972
water–RO interfacial tension, γ (mN/m)	35^50^
oil volume fraction, α_o_	0.15, 0.30, 0.45, 0.60
temperature, *T* (°C)	∼ 22
pressure drop, Δ*P* (kPa)	200
number of passes, *n*	1, 5, 20, 100
Reynolds number (*Re*)	∼ 10000 (based on viscosity of water)
cavitation number (*Ca*)	∼ 1

The emulsion samples were collected from the holding tank at different numbers of passes. The number of passes through the HC device was calculated as *n* = *Qt*/*V*, where *Q* is the flow rate through the HC device, *V* is the total volume of emulsion in the holding tank (and the flow loop), and *t* is the flow time. The DSD values of the collected samples were measured using a Master-sizer 3000 (Malvern Panalytical Ltd. UK) instrument. Considering the intention of investigating dense oil in water emulsions, microscopic images and conductivity measurements were used for identifying the continuous phase of the emulsions. Based on these measurements (see results and discussion included in Section S1 of the Supporting Information), an aqueous phase was confirmed to be the continuous phase for all the emulsions considered in this work. For Master-sizer measurements with the oil droplet and dispersant, the refractive indices of rapeseed oil (1.466) and water (1.33) were used, respectively. The absorption index of the dispersed phase was assumed to be 0.1. The sensitivity of the obscuration level was investigated (see Section S2 of the Supporting Information), and based on these studies it was ensured that the obscuration level for all measurements was between 5% and 10%. Triplicate measurements were carried out with continuous stirring at 2500 rpm.

The turbidity of emulsions was measured in two different ways using a spectrophotometer and turbidimeter. The absorbance values of the emulsions were measured using a SHIMADZU UV-1800 UV–vis spectrophotometer at a wavelength of 630 nm. All measurements were conducted at room temperature using high-precision quartz glass cuvettes (Hellma Analytics 114-10-40) with a light path length of 0.01 m. Notably, these measurements were taken in reference to a baseline solution composed of surfactant-added deionized water. Initially, all emulsion samples were diluted to 1% (v/v) for absorbance measurements. To prevent coalescence during dilution, deionized water with 2% TWEEN 20 surfactant used in the emulsification was used for dilution. Different quantities (0.6, 1.0, 1.4, 1.6, and 1.8 mL) of this 1% diluted emulsion sample were then added to 25 mL of deionized water for absorbance measurements. Turbidity measurements were carried out using a commercial turbidimeter device (VELP Scientifica TB1, Italy) at 25 °C. The turbidimeter used an infrared emitting diode with a wavelength of 850 nm. Here again the emulsion sample diluted to 1% was used for turbidity measurements. Different quantities of diluted sample were added to 25 mL of deionized water with 2% surfactant, and turbidity was measured in NTU (nephelometric turbidity units). All measurements were carried out in triplicate.

## 3 Processing of Experimental Data

### 3.1 Droplet Size Distribution (DSD)

The measured DSDs were represented as sum of three droplet populations (*j* = 1, 2, 3) represented by three log-normal distributions as
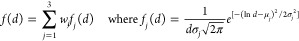
1where *d* is a droplet diameter, *w*_*j*_ is volume fraction of the *j*th log-normal function (LNF), μ_*j*_ is the mean of the *j*th LNF, *f*_*j*_(*d*)Δ*d* is a volume fraction of oil droplets of the *j*th population having diameters between *d* and *d* + Δ*d*, and σ_*j*_^2^ is the variance of *j*th LNF. σ_*j*_ is the standard deviation of the *j*th LNF. The sum of volume fractions of three droplet populations is one.
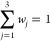
2

The measured DSD was fitted to obtain a set of eight parameters: means (μ_1_, μ_2_, and μ_3_) and standard deviations (σ_1_, σ_2_, and σ_3_) for each of the three distributions and two volume fractions (*w*_1_ and *w*_2_). The nonlinear optimization tool embedded in MS Excel was used to obtained values of these eight parameters by minimizing the sum of square of errors.

**Figure 2:**
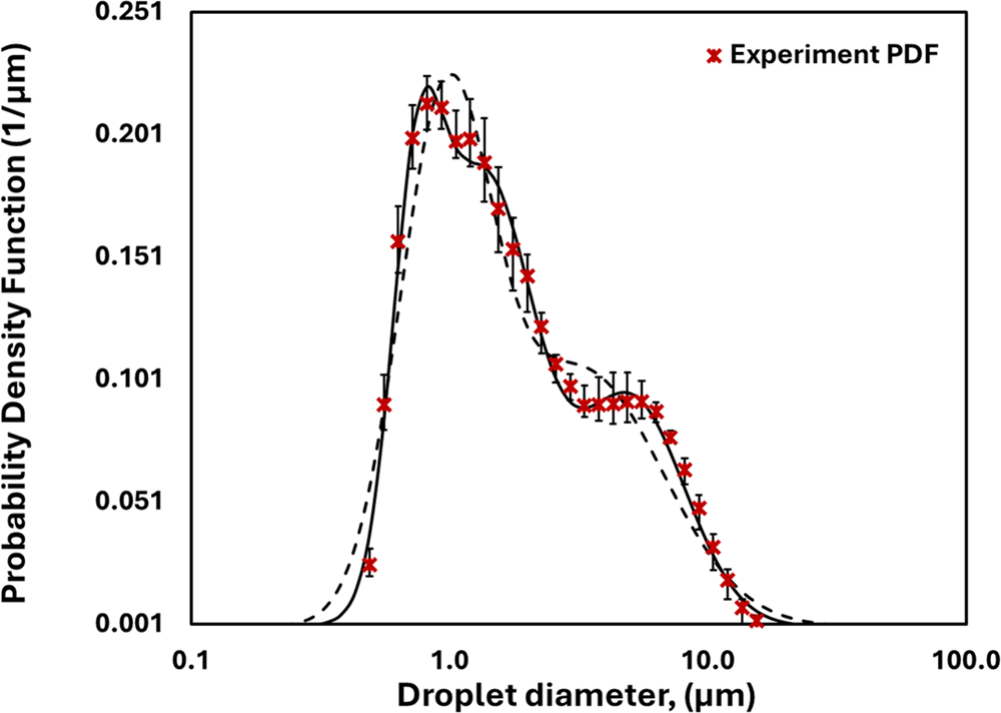
Measured and fitted droplet size distributions for an oil volume fraction of 0.15 (*n* = 100) with three and two log-normal functions (continuuous line, sum of three LNFs; dashed line, sum of two LNFs).

Figure 2 illustrates an example of fitting the measured DSD using eq 1 for the case of a 100-pass emulsion with an oil volume fraction of 0.15. Initially, the DSD was fitted using the sum of two and three LNFs. It can be seen that the sum of three LNFs (eq 1) describes the experimental data quite well. The sum of three LNFs was therefore used subsequently. The measured distributions were also used to calculate a few characteristic droplet sizes such as *d*_43_ (volume-weighted mean diameter) and *d*_32_ (surface-weighted mean diameter) as
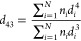
3
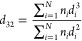
4where *n*_*i*_ is the number of droplets with *d*_*i*_ diameter.

In addition, the emulsion DSD coefficient (also known as span) was calculated as
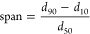
5where *d*_*x*_ is the diameter corresponding to *x* volume % on a cumulative volume distribution curve.

### 3.2 Estimation of Effective Viscosity

The dense oil in water emulsions may exhibit non-Newtonian viscosity. However, in this work, rather than fully characterizing the rheological behavior of a dense oil in water emulsion, the focus was on obtaining the effective viscosity, which will allow a designer to appropriately size the vortex-based hydrodynamic cavitation device for the desired capacity of emulsion production. For this purpose, we used a previously developed generalized correlation relating the pressure drop and flow of Newtonian liquids through the vortex-based HC device used in this work.^51^ The original paper may be referred to for more details. The developed correlation is included in Section S3 of the Supporting Information for ready reference. The measured pressure drop versus flow rate data for emulsions with different volume fractions of oil were used to estimate the effective viscosity. Nonlinear optimization was used to find the effective viscosity for each case by fitting the experimental data using the developed correlation (Section S3 of Supporting Information). The influence of the volume fraction of oil on effective viscosity is discussed in section 4.

### 3.3 Estimation of Effective Diameter from Turbidity/Absorbance

The size of the droplets in a suspension can be estimated by measuring the turbidity of the suspension. Turbidity measures the attenuation of a beam of light traveling through the suspension, which is caused by the scattering and absorption of light by the droplets. The amount of scattering and absorption depends on the sizes of the droplets and their concentration in the suspension. In a standard spectrophotometer, light absorbed by droplets is related to the droplet size. The transmitted light measured by a standard spectrophotometer is related to absorbance as^52−54^
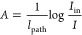
6where *I* is transmitted light intensity and *I*_in_ is the incident light intensity. The log is to base 10. The light transmission path of spectrophotometer cuvettes (*l*_path_) is 0.01 m. The spectrophotometer reports values of absorbance (*A*) for different wavelengths (in units of m^–1^). Unlike the UV spectrophotometer, commercial turbidity meters measure turbidity in NTU by measuring scattered light at 90° to the direction of light beam. The effective turbidity may also be related to detected light intensity (*I*).^55^
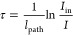
7

The turbidity, τ, measured in NTU by commercial turbidity meters is therefore expected to be proportional to absorbance *A*, measured by the UV spectrophotometer. The turbidity is related to DSD and number density of droplets via theory of light scattering from spherical particles as^55^
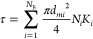
8where *N*_*i*_ is the concentration of the number of droplets of bin *i* (number/m^3^) and *K*_*i*_ is the scattering coefficient for droplets of size *d*_*mi*_. The concentration of droplets is related to the volume fraction of oil in the measurement path (ϵ_*O*_) as
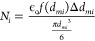
9

Substituting eq 9 into eq 8 leads to
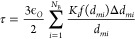
10if the effective ratio of scattering coefficient and diameter is written as
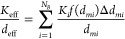
11where *K*_eff_ is an effective scattering coefficient and *d*_eff_ is an effective characteristic droplet diameter. The scattering coefficient attains a value of 2 for droplet diameters much larger than the wavelength of light. Therefore, by setting the value of *K*_eff_ to 2, eq 10 can be simplified as
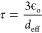
12

Equation 12 was used to process the measured turbidity and absorbance data as a function of the oil volume fraction for estimating the effective characteristic droplet diameter of an emulsion. These results are discussed in section 4.

## 4 Result and Discussion

### 4.1 DSD and Characteristic Droplet Diameters

**Figure 3:**
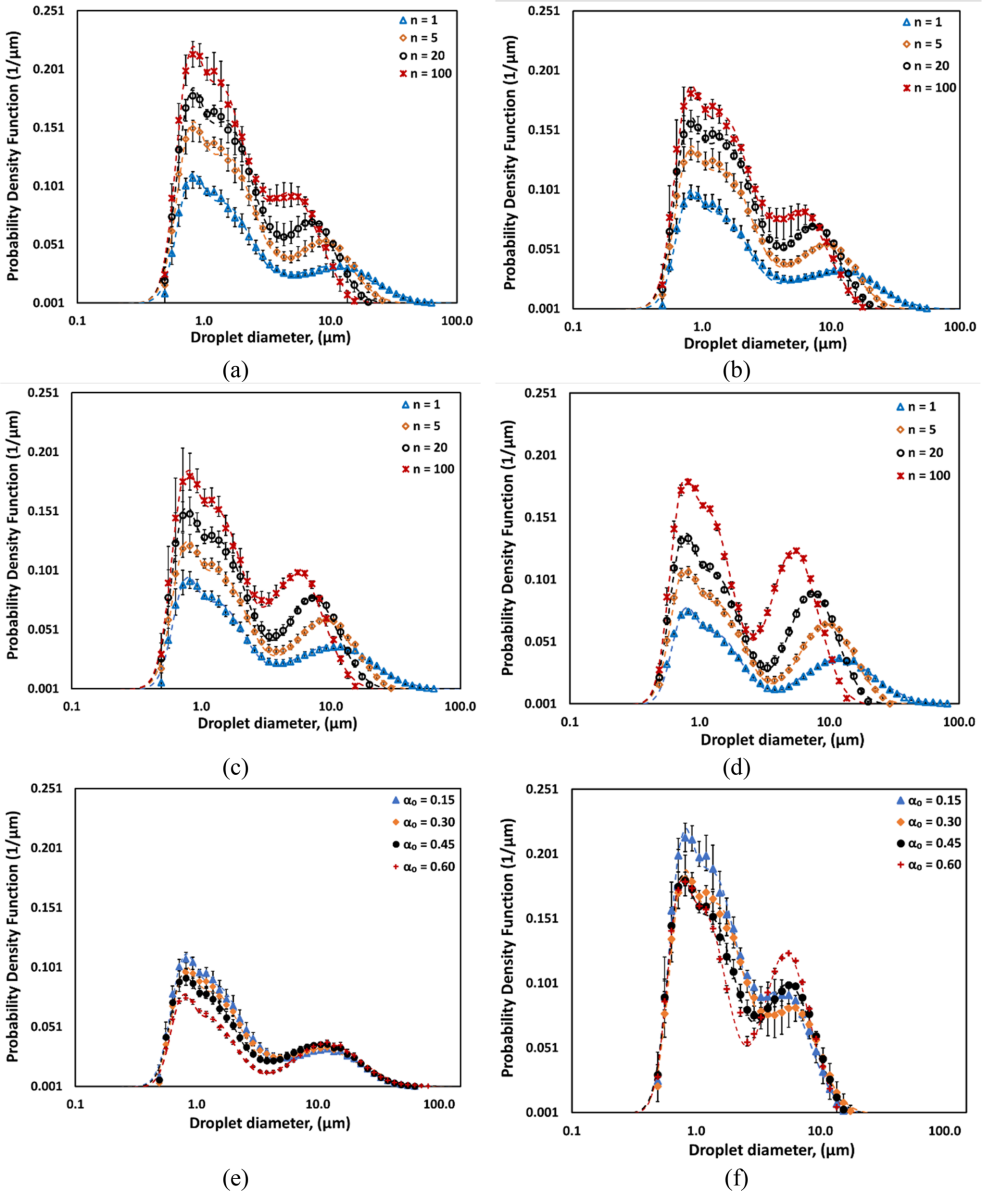
DSD profiles for different rapeseed oil volume fractions. Lines indicate fitted sums of three LNFs: (a) α_o_ = 0.15, (b) α_o_ = 0.30, (c) α_o_ = 0.45, (d) α_o_ = 0.60, (e) *n* = 1, and (f) *n* = 100.

The multiple pass experiments were performed to examine the effect of the oil volume fraction on the droplet size distribution. The measured DSD (volume based) as a function of the number of passes (*n* = 1, 5, 20, and 100) is shown in Figure 3. Figure 3a–d show the influence of the number of passes on DSD for specific oil volume fractions (0.15, 0.30, 0.45, and 0.60 respectively). The influence of the oil volume fraction on the DSD for the number of passes equal to 1 and 100 is shown in Figure 3e and f, respectively. It can be seen that with an increase in the number of passes, the size of droplets decreases and the DSD shifts toward smaller droplet sizes. These DSDs were described by eq 1. The lines shown in Figure 3a–f indicate the DSDs fitted with eq 1. The fit parameters of eq 1 for all of the considered cases are listed in Table 2.

**Table 2:** Three Log-Normal Fitting Parameters for Different Numbers of Passes and Characteristic Droplet Diameters (μm)

*α*_o_	*n*	*W*_1_	*W*_2_	*μ*_1_	*σ*_1_	*μ*_2_	*σ*_2_	*μ*_3_	*σ*_3_	*d*_32_	*d*_10_	*d*_50_	*d*_90_	span
0.15	1	0.03	0.17	–0.22	0.23	0.61	0.49	2.86	0.75	4.89	1.61	13.89	36.34	2.50
	5	0.05	0.28	–0.22	0.23	0.68	0.52	2.41	0.51	3.47	1.28	7.98	17.32	2.01
	20	0.05	0.33	–0.23	0.23	0.66	0.50	2.15	0.45	2.94	1.16	5.81	12.68	1.98
	100	0.06	0.31	–0.24	0.23	0.53	0.46	1.86	0.47	2.55	1.05	4.35	9.62	1.96
0.3	1	0.03	0.15	–0.22	0.22	0.58	0.48	2.84	0.74	5.19	1.74	14.06	34.46	2.35
	5	0.04	0.25	–0.22	0.23	0.66	0.51	2.43	0.52	3.75	1.37	8.61	17.99	1.95
	20	0.04	0.31	–0.24	0.22	0.66	0.50	2.20	0.46	3.18	1.24	6.45	13.55	1.91
	100	0.05	0.32	–0.24	0.23	0.62	0.48	2.00	0.46	2.80	1.14	5.09	11.06	1.95
0.45	1	0.03	0.12	–0.26	0.21	0.51	0.46	2.83	0.73	5.54	1.90	14.26	36.63	2.45
	5	0.03	0.19	–0.26	0.22	0.57	0.49	2.43	0.52	4.12	1.46	9.40	18.77	1.84
	20	0.04	0.23	–0.28	0.21	0.56	0.48	2.20	0.45	3.40	1.28	7.08	13.77	1.77
	100	0.05	0.25	–0.28	0.22	0.49	0.45	1.92	0.46	2.87	1.14	5.28	10.39	1.76
0.6	1	0.10	0.09	–0.25	0.21	0.48	0.44	2.90	0.66	6.70	2.57	16.25	47.07	2.75
	5	0.14	0.14	–0.27	0.22	0.51	0.47	2.47	0.48	4.68	1.66	10.36	19.28	1.70
	20	0.17	0.17	–0.28	0.21	0.47	0.45	2.19	0.43	3.77	1.38	7.66	13.75	1.62
	100	0.19	0.20	–0.30	0.20	0.34	0.40	1.85	0.42	2.94	1.15	5.34	9.59	1.59

**Figure 4:**
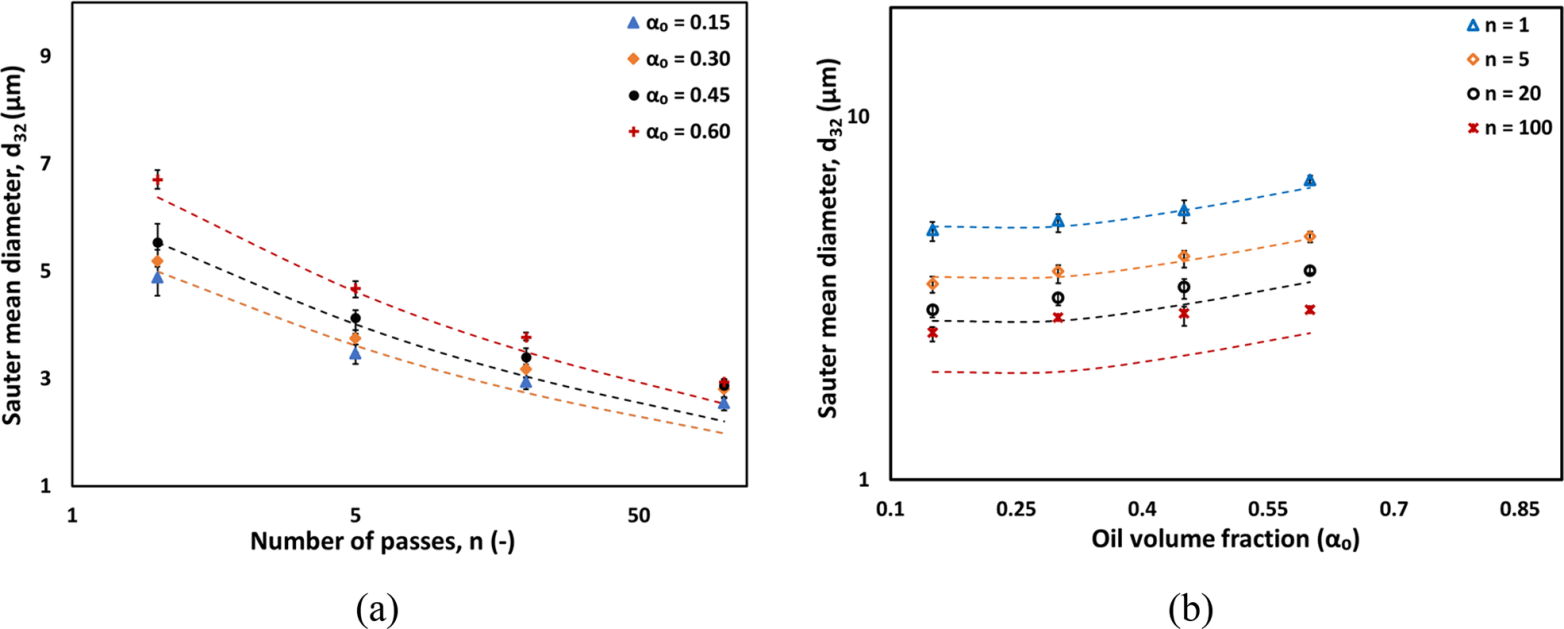
Measured values of the Sauter mean diameter (*d*_32_). Symbols indicate experimental data. (a) Influence of number of passes. Lines indicate *d*_32_ predicted using eq 13. (b) Influence of the oil volume fraction. Lines indicate *d*_32_ predicted using eq 14.

The influence of the number of passes on the Sauter mean diameter is shown in Figure 4a. It can be seen that the Sauter mean diameter (*d*_32_) gradually decreases with an increase in number of passes. For a low oil volume fraction, the dependence of the Sauter mean diameter on number of passes can be expressed as shown by Thaker and Ranade.^20^
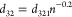
13Here, *d*_321_ is the Sauter mean diameter after the first pass through HC device (*n* = 1).

The influence of the oil volume fraction on the Sauter mean diameter is shown in Figure 4b. It can be seen that the Sauter mean diameter is initially a weak function of the oil volume fraction. However, beyond a certain critical value of oil volume fraction (α_oc_), the Sauter mean diameter increases with a further increase in oil volume fraction. The variation in *d*_321_ as a function of oil volume fraction can thus be approximated using the two regimes defined by a critical oil volume fraction (α_oc_).
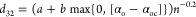
14

The experimental data indicate the values of parameters of eq 14 as *a* = 5, *b* = 5.6, and α2 = 0.35. Here *d*_32_ is independent of the oil volume fraction (α_o_) if it is less than the critical oil volume fraction (α_oc_). For oil volume fractions higher than α_oc_, *d*_32_ is linearly proportional to the excess oil volume fraction beyond 0.35 (that is, [α_o_ – α_oc_]).

**Figure 5:**
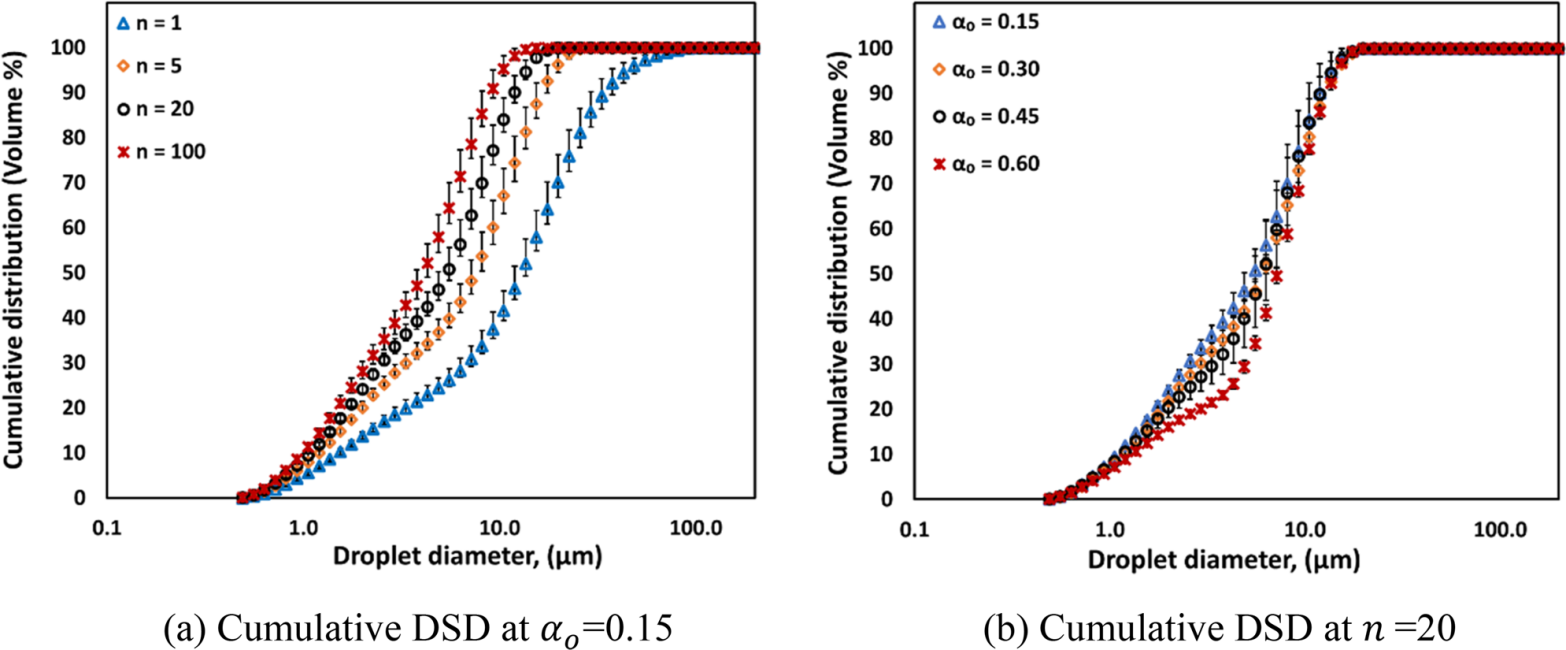
Influence of the number of passes and oil volume fraction on the cumulative DSD.

It will be instructive to examine other characteristic droplet diameters such as *d*_10_, *d*_50_, and *d*_90_. Cumulative droplet size distributions were therefore examined. As an example, the influence of the number of passes on cumulative distributions for the case of an oil volume fraction of 0.15 is shown in Figure 5a. These cumulative DSDs were then used to calculate characteristics droplet diameters *d*_10_, *d*_50_, and *d*_90_. The cumulative profiles suggest that with an increase in the number of passes there is a higher rate of breakage for larger droplets compared to smaller droplet sizes. A similar trend was observed across various emulsions with different oil volume fractions of 0.30, 0.45, and 0.60. The characteristic droplet diameters (*d*_10_, *d*_50_, and *d*_90_) for each oil volume fraction as a function of number of passes are listed in Table 2. Further, the influence of the oil volume fraction on the cumulative DSD is shown in Figure 5b for the number of passes *n* = 20. The influence of the oil volume fraction becomes apparent at higher values of oil volume fractions (0.45 and 0.6).

**Figure 6:**
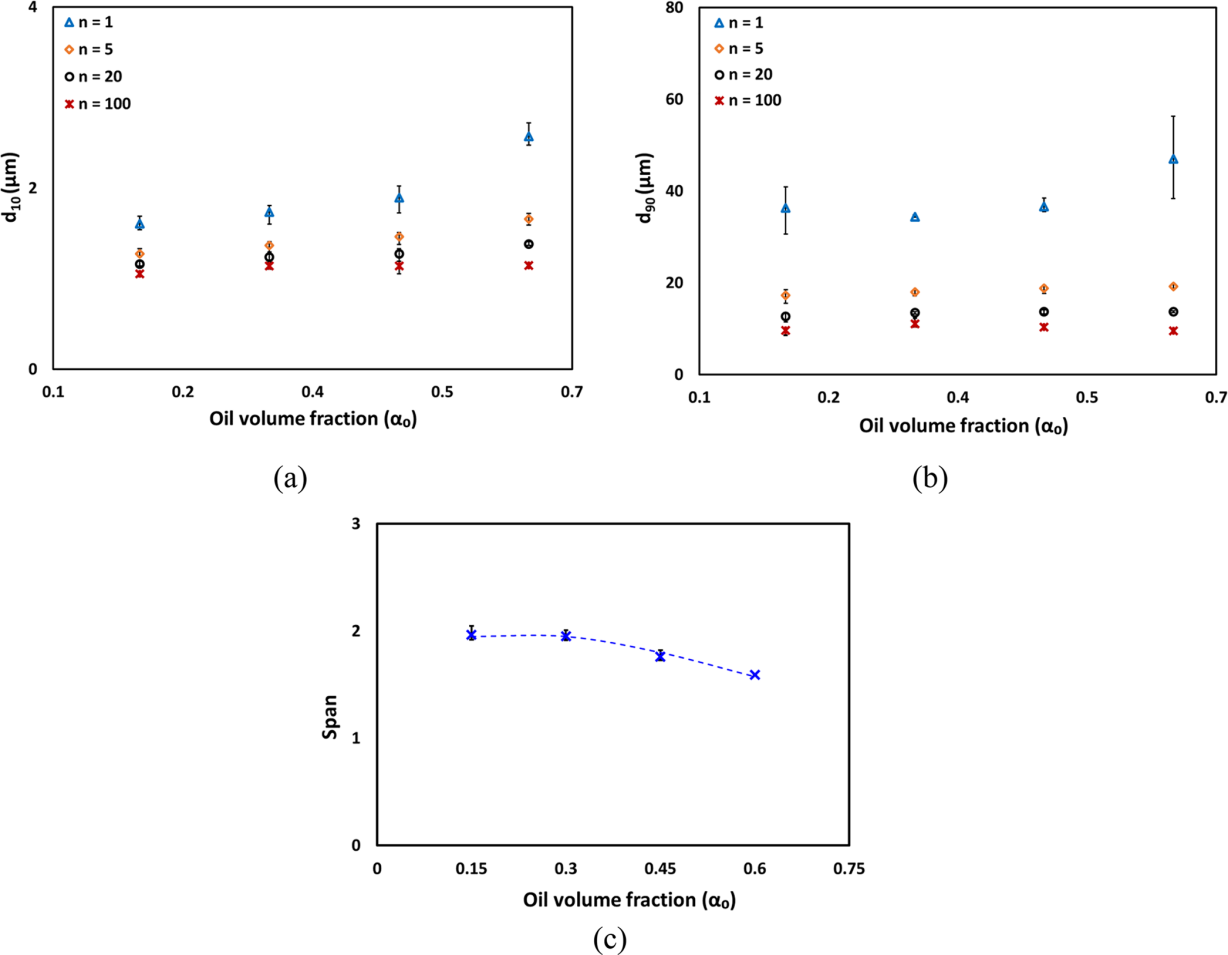
Effect of the oil volume fraction and number of passes on characteristic droplet diameters (a) *d*_10_ and (b) *d*_90_. (c) Span for *n* = 100 passes.

The influence of the oil volume fraction on values of *d*_10_ and *d*_90_ is shown in Figure 6a and b, respectively. It can be seen that *d*_10_ follows trends similar to *d*_32_ showing almost no influence from the oil volume fraction until α_o_ = 0.30; beyond that, it increases with the oil volume fraction. In contrast, for *d*_90_, the oil volume fraction has almost no influence even up to α_o_ = 0.6 for *n* ≥ 5. As the number of passes through the cavitation device increases, larger droplets are easily broken into smaller ones. Therefore, within first few passes, the value of *d*_90_ decreases sharply and remains same for subsequent increase in number of passes. Smaller droplets are harder to break and therefore require a greater number of passes through the cavitation device to become independent of the number of passes. The higher the volume fraction of oil, the greater number of passes are needed (see Figure 6a). All characteristic droplet diameters (*d*_10_–*d*_90_) for emulsions characterized in this work are listed in Table S1 of the Supporting Information. The variation of characteristic values of emulsion DSD coefficient or span  as a function of oil volume fraction for *n* = 100 are shown in Figure 6c. It can be seen that the behavior of span with the oil volume fraction also exhibits two regimes and may be represented as
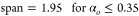
15
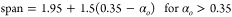
16

### 4.2 Effective Viscosity of Emulsion

**Figure 7:**
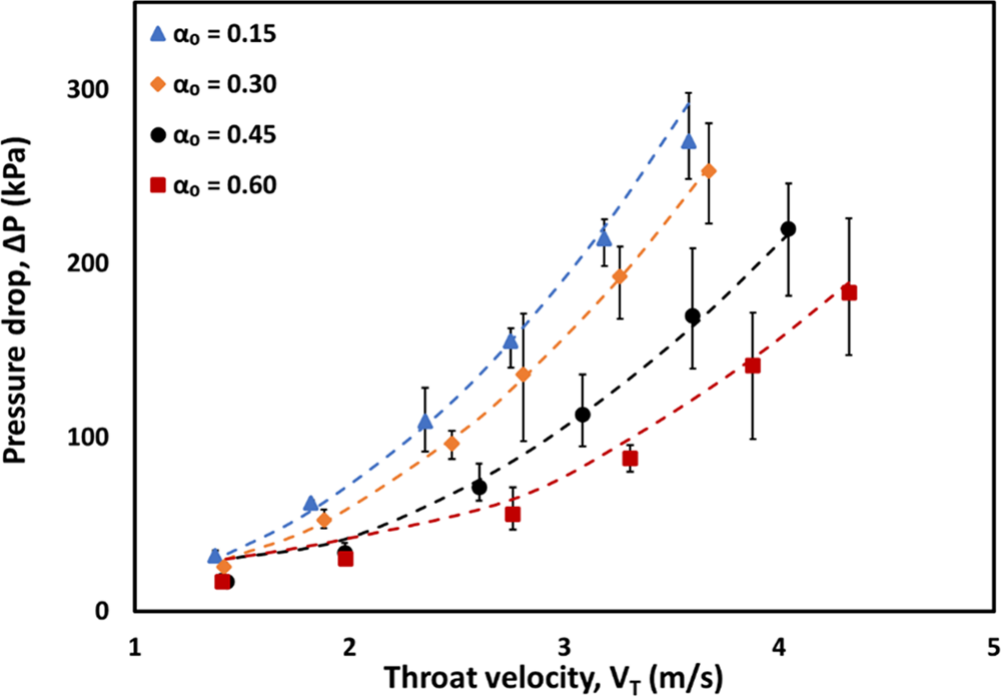
Fitted and measured pressure drop versus throat velocity for different oil volume fractions: (a) α_o_ = 0.15, μ_eff_ = 0.97 mPa·s; (b) α_o_ = 0.30, μ_eff_ = 1.6 mPa·s; (c) α_o_ = 0.45, μ_eff_ = 4.2 mPa·s; and (d) α_o_ = 0.60, μ_eff_ = 9.0 mPa·s . Symbols denote experimental data, and dashed lines of the same color indicate fitted results based on the correlation of Thaker et al.^51^ by adjusting effective viscosity.

The influence of the oil volume fraction (α_o_) on the effective viscosity was characterized by measuring flow characteristics (pressure drop versus flow rate) of the emulsion through a HC device. As mentioned in section 2, emulsions generated after 100 passes were used for this purpose. The measured pressure drop values at different flow rates are shown in Figure 7 (in terms of throat velocity, *V*_T_). As expected, the measured pressure drop increases with an increase in flow rate (or increase in throat velocity, *V*_T_) for all cases. However, it is interesting to note that for the same flow rate the measured pressure drop was found to decrease with an increase in the oil volume fraction of emulsion. This may appear counterintuitive, since a higher volume fraction of oil leads to an increase in viscosity. This apparent counterintuitive behavior can be explained by the three distinct regimes in the Euler number versus Reynolds number relationship exhibited by vortex-based HC devices.^51^ As the volume fraction of oil increases, the effective viscosity increases. For the same flow rate, this leads to a reduction in the Reynolds number and therefore a reduction in the Euler number, leading to a reduced pressure drop with an increase in the oil volume fraction. The Euler number  of a vortex-based hydrodynamic cavitation device was found to decrease with an increase in the volume fraction of oil.

**Figure 8:**
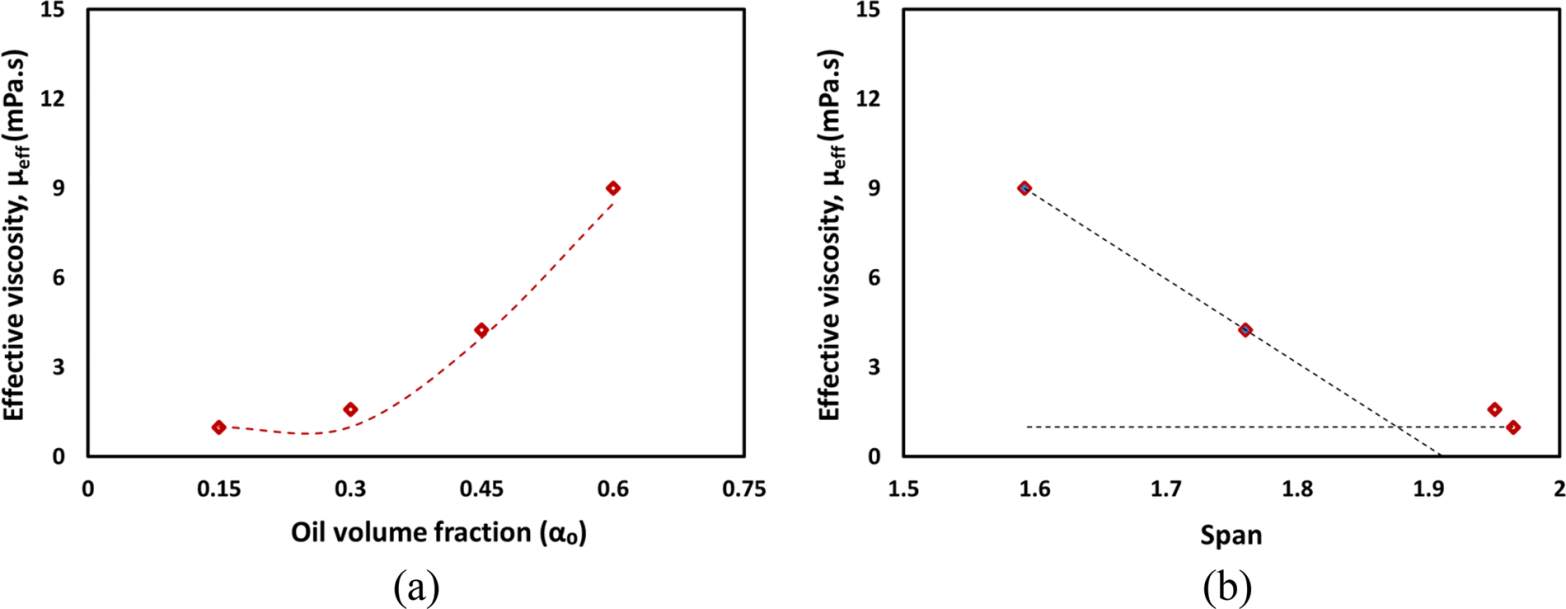
Influence of the oil volume fraction and span on the effective viscosity of emulsions (*n* = 100). Dashed lines indicate the trends.

The measured pressure drop data was used to estimate the effective viscosity of the emulsion using the correlations developed in our previous work (Thaker et al.^51^). The nonlinear optimization and correlation of Thaker et al. was used to obtain fitted values of effective viscosity. The fitted results show good agreement with the experimental data (see Figure 7). The estimated effective viscosities for emulsions with different oil volume fraction values are included in the figure caption. The estimated viscosity of emulsion with varying oil volume fraction (α_o_) under same pressure drop condition is shown in Figure 8. As expected, higher viscosities were exhibited by emulsions with higher oil volume fractions (see Figure 8a). An increase in the oil volume fraction from 0.15 to 0.6 leads to an increase in effective viscosity by almost an order of magnitude (from 0.97 to 9 mPas). As we have observed earlier, oil volume fraction of 0.35 is a transition point between two regimes. For effective viscosity **(**μ_eff_), the following relationships were found to hold:
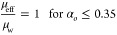
17
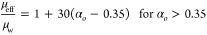
18where μ_w_ is the viscosity of water.

The results also indicate that the smaller the value of span, the higher the viscosity (see Figure 8b). Similar observation was also reported by Mugabi and Jeong.^35^ For emulsions with larger span, small droplets coexist with larger droplets. Smaller droplets may act as a lubricant and reduce the effective viscosity. However, as the value of span decreases, such a lubricating action of smaller droplets becomes less effective, leading to higher values of effective viscosity.

### 4.3 Turbidimetric Analysis

**Figure 9:**
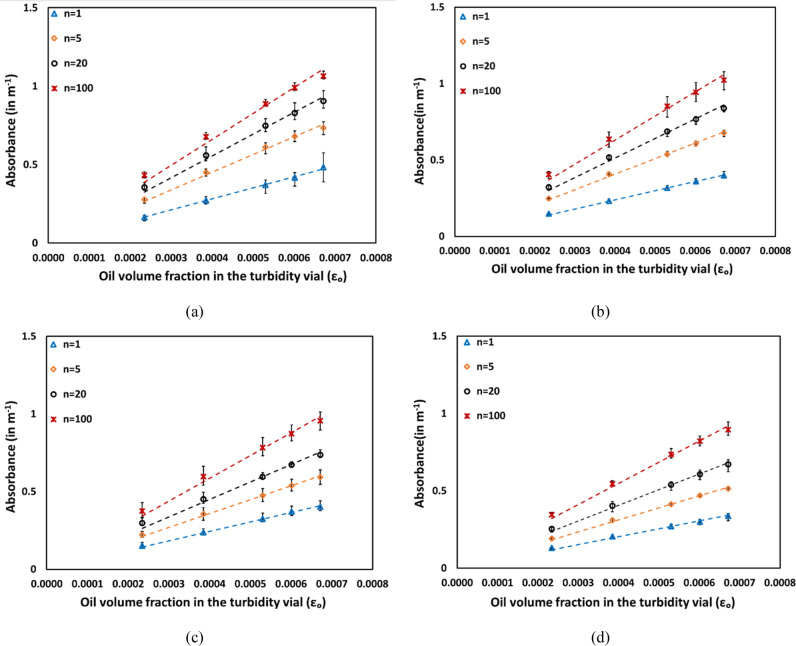
Absorbance for emulsions with different numberes of passes through the HC device: (a) α_0_ = 0.15, (b) α_o_ = 0.30, (c) α_o_ = 0.45, and (d) α_o_ = 0.60.

Visible light absorbance data were obtained for emulsion oil volume fractions α_o_ = 0.15, 0.30, 0.45, and 0.60 at a wavelength of 630 nm. The measured values of absorbance as a function of the oil volume fraction in the measurement vial (ϵ_o_) containing emulsions obtained for different number of passes are shown in Figure 9. Emulsions obtained at different numbers of passes for different oil volume fractions have different Sauter mean diameters (*d*_32_), as listed in Table 2. As expected, the absorbance increases linearly with the increase in oil volume fraction for emulsions obtained with a particular number of passes. Moreover, it is evident that emulsions with a higher number of passes, for the same oil volume fraction in the vial, exhibit higher absorbance, as seen in Figure 9a–d. The observed differences in absorbance among emulsions in relation to the number of passes are directly linked to variations in characteristic droplet size. In our experimental setup, emulsions were obtained at various numbers of passes, and oil droplets underwent breakage when repeatedly exposed to the cavitating zone in the vortex-based HC device. As a result, with a constant oil volume fraction, an increase in the number of passes results in further breakage of oil droplets into smaller sizes. Consequently, emulsions obtained with a higher number of passes exhibit higher number density of droplets, contributing to an overall increase in absorbance. As discussed in section 4.1, larger droplets were observed in emulsions with higher oil volume fractions. Therefore, the absorbance was found to decrease as the oil volume fraction increased from 0.15 to 0.60 for the same number of passes (see Figure 9a–d).

**Figure 10:**
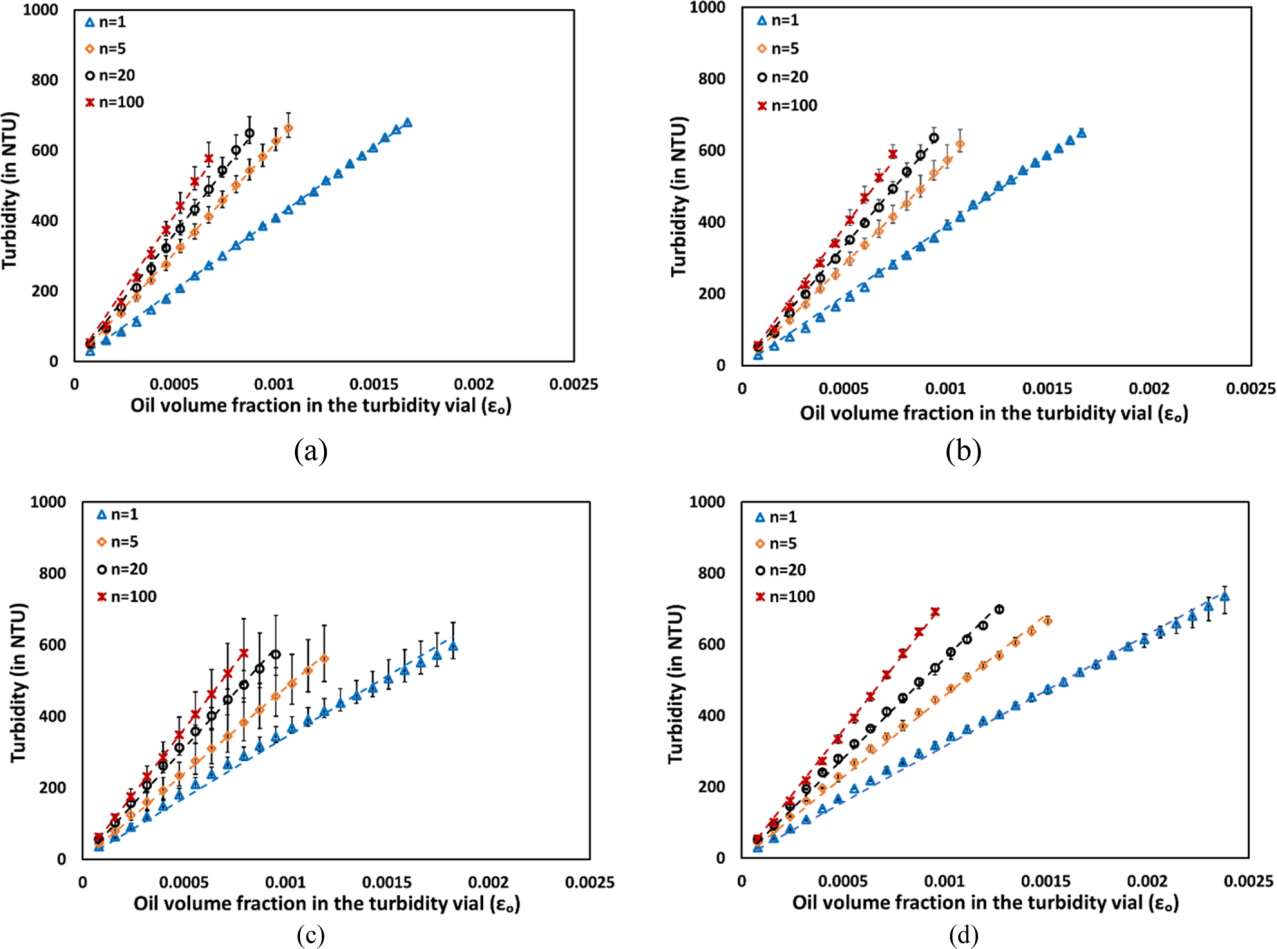
Turbidity measurements for emulsions with different numberes of passes through the HC device: (a) α_0_ = 0.15, (b) α_o_ = 0.30, (c) α_o_ = 0.45, and (d) α_o_ = 0.60.

The turbidity data measured in NTU using a turbidity meter for emulsions with four different oil volume fractions (α_o_ = 0.15, 0.30, 0.45, and 0.60) and various numbers of passes (*n* = 1, 5, 20 and 100) are shown in Figure 10. The observed turbidity profiles also exhibit a linear relationship with respect to the oil volume fraction in the measurement vial (ϵ_o_). Similar to the absorbance findings (Figure 9), the turbidity results indicate that emulsions characterized by larger droplets (lower number density) consistently display lower turbidity (in NTU) compared with those featuring smaller droplets (higher number of passes). As discussed in section 3.3 and eq 12, the rate of change of turbidity or absorbance with respect to the oil volume fraction in the measurement vial (ϵ_o_) is inversely proportional to the effective characteristic droplet diameter (*d*_eff_) of emulsions. Therefore, the slopes of absorbance and turbidity with respect to oil volume fraction in vial (ϵ_o_) were calculated and examined.

The slopes of absorbance (in m^–1^) and turbidity (in NTU) with respect to oil volume fraction were related as follows (see Figure S6 in Section S4 of the Supporting Information):
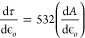
19Here, *A* is absorbance at 630 nm wavelength (in m^–1^) and τ is turbidity in NTU. Because of this linear relationship between turbidity and absorbance, either of these parameters can be employed to estimate the effective characteristic diameter (*d*_eff_) using eq 12 and eq 19 as
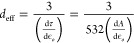
20

Using eq 20 and measured turbidity data, the effective diameters, *d*_eff_, for emulsions of different oil volume fractions and number of passes were calculated. The effective diameter, *d*_eff_, was found to closely mimic the behavior of and be linearly related to *d*_32_. The relationship between the effective diameter estimated using eq 20 and the turbidity data with the Sauter mean diameter may be represented as follows (see Figure S7 of the Supporting Information):
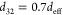
21

**Figure 11:**
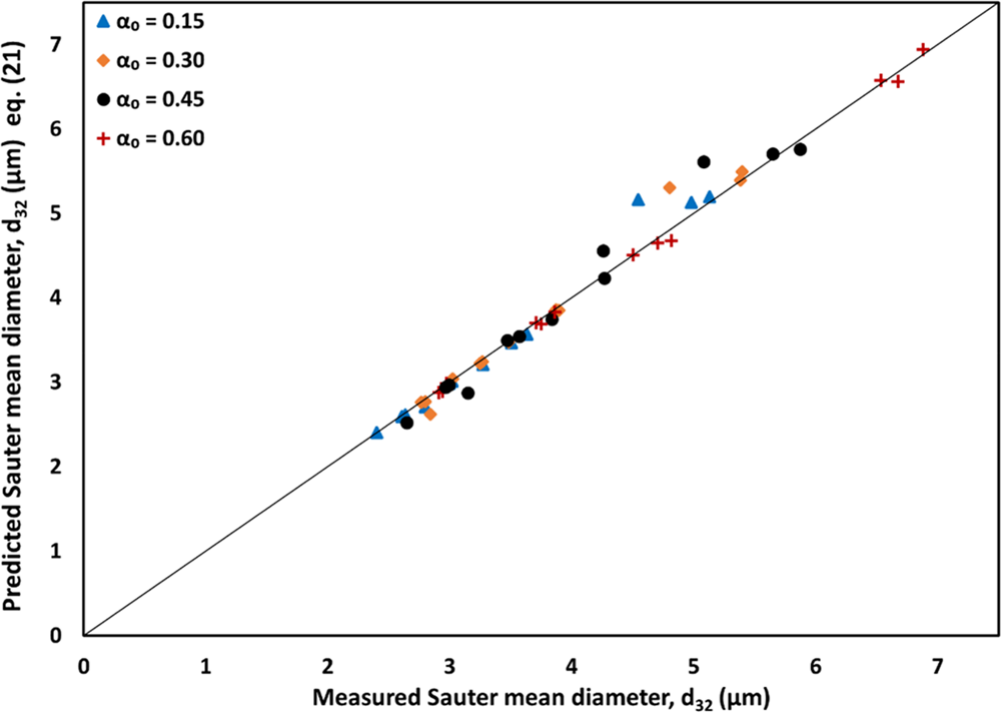
Comparison of the Sauter mean diameter (*d*_32_) obtained from Master-sizer with that estimated from turbidity measurements and eqs 20 and 21).

The comparison between the predicted Sauter mean diameter using eqs 20 and 21 and the values obtained from the Master-sizer-measured DSD are shown in Figure 11 in the form of a parity plot. It can be seen from Figure 11 that the turbidity (or absorbance) data can provide adequately accurate estimations of the Sauter mean diameter, *d*_32_, for emulsions with different oil volume fractions obtained with different numbers of passes. Turbidity measurements thus offer the potential to use just single-point data for estimating characteristic droplet diameter and *d*_32_ if the slope can be reliably estimated from the single point measurement of turbidity. The presented approach and data will be useful for further work on the development of quick methods for estimating characteristic droplet diameter of dense emulsions.

### 4.4 Droplet Breakage Efficiency

The knowledge of Sauter mean diameters allows the calculation of the droplet breakage efficiency, η. Thaker at al.^57^ previously reported the droplet breakage efficiency of a vortex-based HC device for low oil volume fractions. For quantifying influence of higher oil volume fraction on droplet breakage efficiency, the data collected in this work were used to calculate droplet breakage efficiency following the method from Thaker and Ranade^20^ as
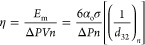
22Here, *E*_m_ is theoretical minimum energy required for drop breakage, with further details provided in the work of Thaker and Ranade^20^ and the references therein. It is useful to compare the η for different oil volume fractions based on energy consumption per unit mass of emulsion *E*, which can be related to pressure drop as
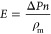
23

**Figure 12:**
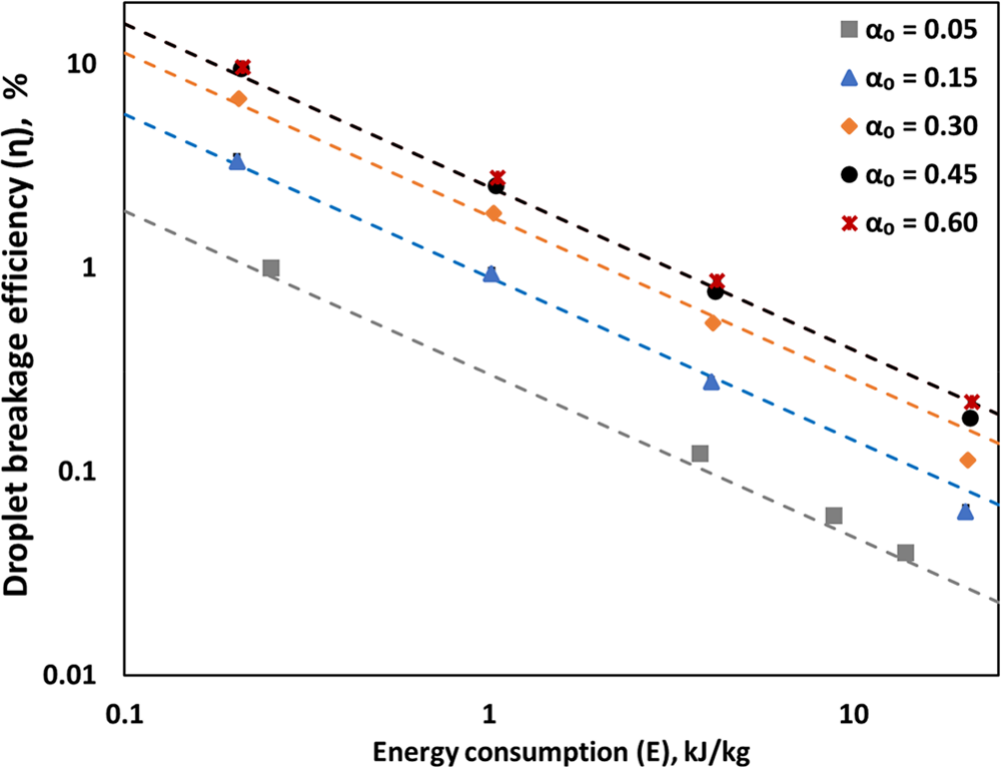
Droplet breakage efficiency (η) as a function of energy consumption Δ*P* = 200 kPa; dashed lines indicate η predicted using eqs 24 and 25.

The droplet breakage efficiency (η) values of the vortex-based HC device for different oil volume fraction emulsions as a function of energy consumption per unit mass (*E*) are shown in Figure 12. The values of η of vortex-based HC device for an oil volume fraction of 0.05 (α_o_ = 0.05), as reported by Thaker and Ranade,^57^ are also included for comparison in Figure 12.

It can be seen that initially the breakage efficiency was found to increase with increase in oil volume fraction. However, there is hardly any difference in the breakage efficiencies obtained for oil volume fractions of 0.45 and 0.6. This is obvious by considering the trends of Sauter mean diameter with the oil volume fraction discussed earlier. Following similar trends, the dependence of droplet breakage efficiency on oil volume fraction can also be represented by the following two equations:
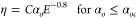
24
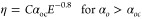
25

The value of parameter *C* in these equations was found to be 6. Up to certain critical oil volume fraction (α_*oc*_, which is ∼0.35 for the considered oil–water–surfactant system), the Sauter mean diameter is independent of the oil volume fraction and therefore the droplet breakage efficiency increases linearly with an increase in oil volume fraction (see eq 24). Beyond the critical oil volume fraction, the Sauter mean diameter was found to increase with a further increase in the oil volume fraction. The droplet breakage efficiency therefore becomes independent of the oil volume fraction in this regime (eq 25). The dependence with respect to energy consumption per unit mass of emulsion was found to be the same over the entire range of oil volume fractions studied here (up to 60% oil in water). The breakage efficiency was found to decrease with increase in energy consumption per unit mass and was found to be proportional to *E*^–0.8^. This is consistent with the results reported by Thaker and Ranade.^20^

## 5 Conclusions

Oil in water emulsions were prepared using a vortex-based HC device. These emulsions comprised a continuous phase of water with Tween 20 surfactant and various volume fractions of rapeseed oil (0.15, 0.30, 0.45, and 0.60). The emulsion samples were collected at 1, 5, 20, and 100 passes for subsequent analysis. The DSD values were measured using laser diffraction techniques. The effective viscosity of the emulsions was measured using the data of pressure drop at different flow rates and a previously published correlation. Using the measured DSD, the Sauter mean diameter, key characteristic diameters, span, and droplet breakage efficiency values were calculated for each case. The key conclusions based on the present work are as follows:

- The cavitation device considered in this work was found to result in bimodal DSD, particularly for small number of passes through the device. As number of passes through the device increases, the DSD approaches a unimodal nature.
- The Sauter mean diameter was found to be independent of oil volume fraction up to certain critical oil volume fraction (α_*oc*_). Beyond this critical volume fraction, the Sauter mean diameter was found to increase with further increases in the oil volume fraction. For the oil–water system considered in this work, this critical oil volume fraction was found to be ∼0.35.
- The other key emulsion characteristics such as effective viscosity, span, and droplet breakage efficiency also similarly exhibit two regimes separated by the critical oil volume fraction.
- For emulsions with oil volume fraction less than α_oc_, viscosity and span are almost independent of oil volume fraction. Beyond this critical oil volume fraction, viscosity increases while span decreases with further increases in the oil volume fraction.
- The rate of change of turbidity (measured in NTU) or absorbance (measured in m^–1^) with respect to the oil volume fraction can be used to estimate the Sauter mean diameter of emulsions.
- Droplet breakage efficiency is linearly proportional to oil volume fraction up to the critical oil volume fraction (α_oc_), beyond which it becomes independent of oil volume fraction.

The presented data show interesting features of dense oil in water emulsions. The turbidity or absorbance measurements may offer a way forward for developing a quick method for estimating characteristic diameters of emulsions, such as the Sauter mean diameter. The presented results will be useful for characterizing dense emulsions and harnessing vortex-based cavitation devices for producing desired dense emulsions.

## Supporting Information Available

Identification of the continuous phase, sensitivity of the obscuration level in laser diffraction measurements, correlations for estimating pressure drop, correlations between turbidity and absorbance, and correlation between the Sauter mean diameter and the effective diameter (PDF)

## Nomenclature

*A*: absorbance (1/m)
*Ca*: cavitation number (−)
*d*_43_: volume averaged bubble diameter (μm)
*d*_32_: Sauter mean diameter (μm)
*d*_*x*_: diameter of the *i*th droplet (μm)
*d*_90_: droplet diameter at 90 vol % in the DSD curve (μm)
*d*_10_: droplet diameter at 10 vol % in the DSD curve (μm)
*d*_50_: median of volume distribution or 50 vol % in the DSD curve (μm)
*d*_T_: throat diameter (mm)
*d*_eff_: droplet effective diameter (m)
*E*: energy consumption per unit mass of emulsion (J/kg)
*Eu*: Euler number (−)
*I*: transmitted light intensity (−)
*I*_in_: incident light intensity (−)
*K*: light scattering coefficient
*l*_path_: path length of the light (m)
*m*: refractive index ratio (−)
*N*_*i*_: concentration of particle
*n*: number of passes (−)
Δ*P*: inlet pressure drop (kPa)
*P*_2_: downstream pressure (kPa)
*P*_v_: vapor pressure (kPa)
*Q*: volumetric flow rate (m^3^/s)
*Re*: Reynolds number, (−)
*T*: temperature (°C)
*t*: time (s)
*V*: volume (m^3^)
*V*_*T*_: throat velocity (m/s)

## Greek Letters

α_o_: oil volume fraction, (−)
ρ: density (kg/m^3^)
μ: viscosity (mPa.s)
γ: interfacial tension (mN/m)
η: energy efficiency for droplet breakage (%)
μ_w_: water viscosity (mPa·s)
τ: turbidity (1/m)
ε_o_: oil volume fraction in the turbidity vial (−)

## Acronyms

DSD: droplet size distribution
HC: hydrodynamic cavitation
LNF: log-normal function
NTU: nephelometric turbidity units
PDF: probability density function
RO: rapeseed oil
VD: vortex-based cavitation device
