# Supplementary material for: Dense Oil in Water Emulsions using Vortex-Based Hydrodynamic Cavitation: Effective Viscosity, Sauter Mean Diameter, and Droplet Size Distribution
Source: Ind Eng Chem Res. 2024 Mar 11;63(11):4977–90. doi: 10.1021/acs.iecr.3c04555 (PMC10958511; doi:10.1021/acs.iecr.3c04555)
Supplement: Supplementary file 1 — ie3c04555_si_001.pdf [file ie3c04555_si_001.pdf]

Supplementary Information

Dense Oil in Water Emulsions using Vortex-based Hydrodynamic Cavitation:

Effective viscosity, Sauter mean diameter and droplet size distribution

Mukesh Upadhyay, Akshay Ravi and Vivek V. Ranade*
Multiphase Reactors and Intensification Group
Bernal Institute, University of Limerick, Limerick V94T9PX, Ireland
*Email: vivek.ranade@ul.ie

Section S1: Identification of continuous phase

The continuous phase of the emulsion is not dictated by the volume fraction alone. It depends on variety of factors including the way emulsions are prepared. In the present case, the continuous phase of the emulsions was confirmed via microscopic images as well as via conductivity measurements. Emulsions with water as a continuous phase exhibit much higher conductivity than that with oil as a continuous phase. Typical microscopic images of emulsions studied in this work are shown in Figure S1. For the sake of brevity, only the images of oil volume fraction of 0.60 (which is oil dispersed in water) and oil volume fraction of 0.95 (which is water dispersed in oil) are shown:

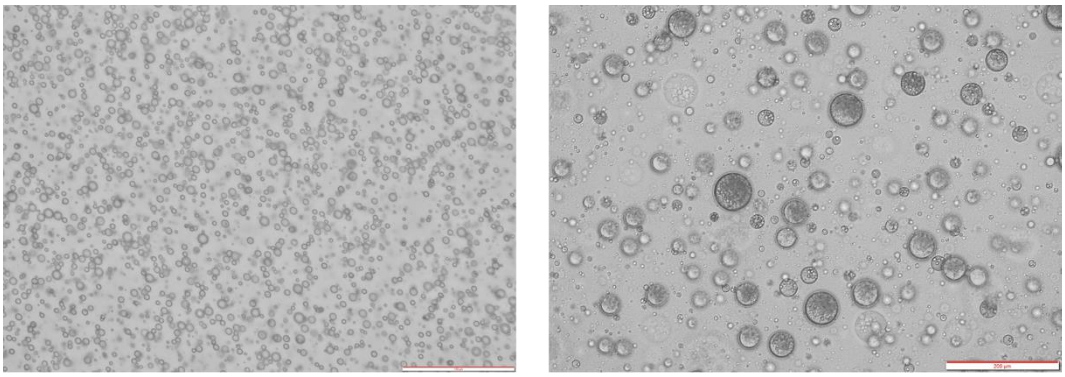

Oil volume fraction, $\alpha_o - 0.6$ (oil-in-water) Oil volume fraction, $\alpha_o - 0.95$ (water-in-oil)

Figure S1. Microscopic images of emulsions.

It can be seen that for 0.95 oil volume fraction emulsion, droplets of oil are darker, which are distinguishable from the droplets seen in 0.60 oil volume fraction (and lower) emulsion. The conductivity values of emulsions with varying oil volume fractions are shown in Figure S2. The conductivity values of pure rapeseed oil and water with 2% Tween 20 surfactants are indicated by dotted lines in Figure S2.

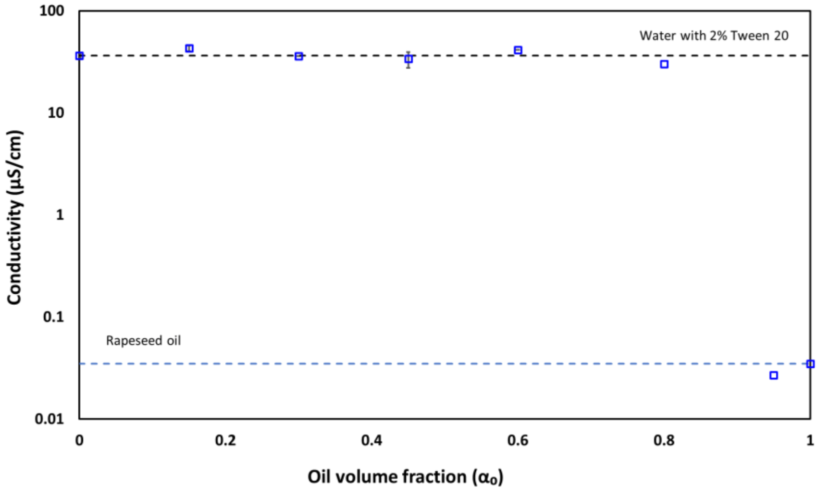

Figure S2: Measured conductivity values of emulsions.

It can be seen from Figure S2 that for all the emulsions considered in this work (oil volume fraction up to 0.60), the measured conductivity values are very close to the conductivity values of surfactant containing water, indicating that the continuous phase is aqueous phase. For oil volume fraction of 0.95 emulsion (which is 0.05 water volume fraction dispersed in oil), the measured conductivity value is close to that of pure rapeseed oil, indicating that oil is the continuous phase. The exact point of phase inversion was not quantified in this work.

For establishing adequate surfactant quantify to be used in the emulsion experiments, the quantity of surfactant needed for mono-layer coverage was first estimated. For the highest oil volume fraction (0.60) considered in this study, even if the Sauter mean diameter is one micron, the quantity of Tween 20 surfactant required for forming a monolayer on all oil drops is less than 0.1% (wt). We used significantly excess surfactant (2 wt%) for all our experiments to ensure that there is no influence of surfactant quantity used on measured droplet size distributions (DSD). Preliminary experiments were carried out to examine influence of surfactant quantity on measured DSD. With the use of 2% Tween 20 surfactant, produced emulsions were found to be stable for a long time. The measured DSDs for oil volume fraction of 0.60 emulsion with the gap of 90 days are shown in Figure S3.

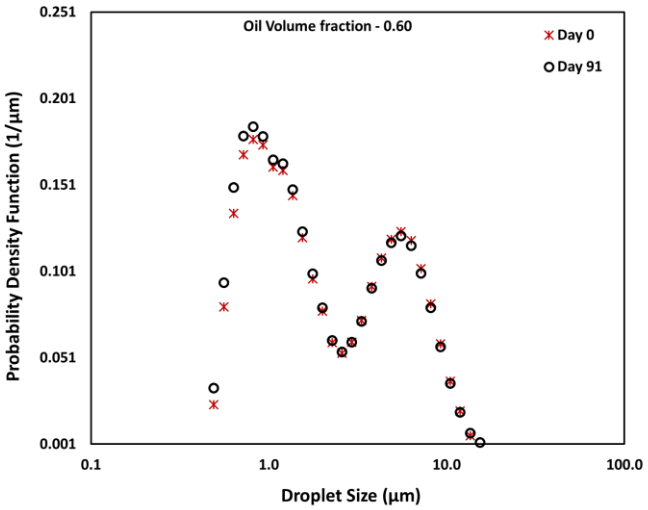

Figure S3: Influence of time elapsed on measured DSD for 60% oil in water emulsion.

It can be seen from Figure S3 that the measured DSDs do not change significantly at least up to 90 days. For all the experimental results reported in this manuscript, the DSD measurements were carried out within a day after the emulsions were produced. The used quantity of surfactant can therefore be considered as adequate.

Section S2: Sensitivity of obscuration level in Laser diffraction measurements:

In Malvern Mastersizer 3000 utilize obscuration, the measure of light blocked or scatter by droplets, to gauge droplet concentration within the measurement cell. With increase in sample volume results in higher laser obscuration. Therefore, ensuring the appropriate range of obscuration is important for accurate measurements. The lower limit is set where the signal-to-noise ratio ensures reproducibility, while the upper limit is influenced by the occurrence of multiple scattering. To establish the upper limit to prevent the multiple scattering we conducted measurements of the same sample at various obscuration levels. Subsequently, we employed a three- log-normal function to fit the Probability Density Function (PDF) of the volume distribution.

$$f(d) = \sum_{j=1}^{3} w_j f_j(d) \text{ where } f_j(d) = \frac{1}{d\sigma_j\sqrt{2\pi}} e^{\left[-\frac{(\ln d - \mu_j)^2}{2\sigma_j^2}\right]} \quad \text{(S-1)}$$

The measured DSD and fitted parameters are shown in Figure S4:

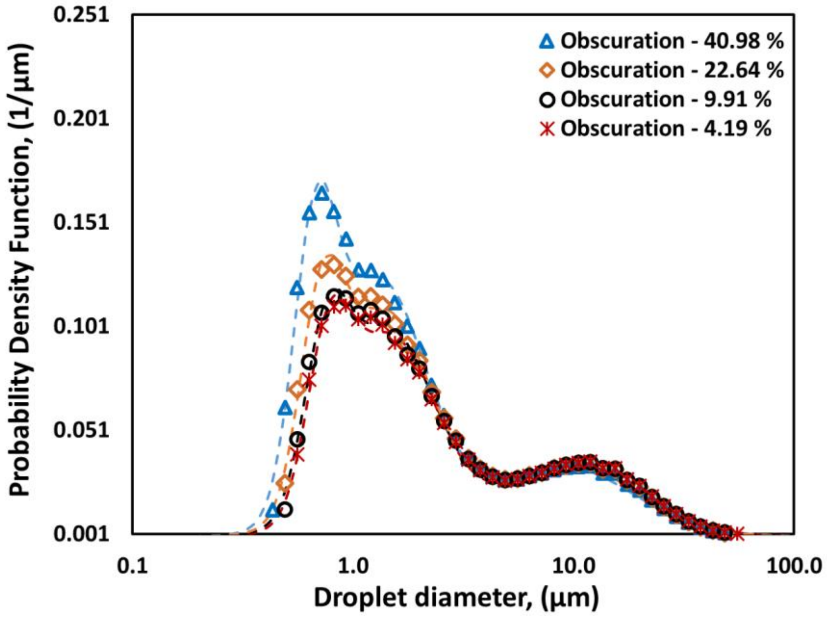

Obscuration (%)	W_1	W_2	μ_1	σ_1	μ_2	σ_2	μ_3	σ_3
4.19	0.031	0.191	-0.209	0.221	0.612	0.484	2.767	0.695
9.91	0.031	0.199	-0.230	0.225	0.603	0.495	2.761	0.700
22.64	0.036	0.209	-0.278	0.223	0.572	0.495	2.745	0.725
40.98	0.046	0.226	-0.348	0.223	0.522	0.496	2.730	0.740

Figure S4: DSD obtained from Mastersizer for oil volume fraction (α_o) of 0.15 at n =1 pass, with different obscuration values (%). The symbols represent the measured values, and the corresponding-coloured lines represent the fitted three log normal distribution obtained from Equation S-1.

It can be seen from Figure S4 that DSD obtained with obscuration level between 4% and 10% are similar. At higher than 10% obscuration levels, the DSDs become wider, as evidenced by the increased σ values in the fitted log-normal distributions. To further illustrate the influence of obscuration levels, we present the standard deviation analysis for small droplets, at varying obscuration levels, as depicted in Figure S5. As outlined in Section 3.1, the acquired PDFs were fitted using three log-normal functions representing distinct droplet size ranges, namely PDF-1, PDF-2, and PDF-3, from smaller droplets to larger droplets. The standard deviation for PDF-1 is shown in Figure S5. It can be seen that standard deviation remained relatively constant until an obscuration level of approximately 10%. Beyond this level, a decline was observed, signifying the escalating influence of multiple scattering. Consequently, to ensure data accuracy and prevent artificial peak induced by multiple scattering, all measurements were conducted within the range of 5-10% laser obscuration.

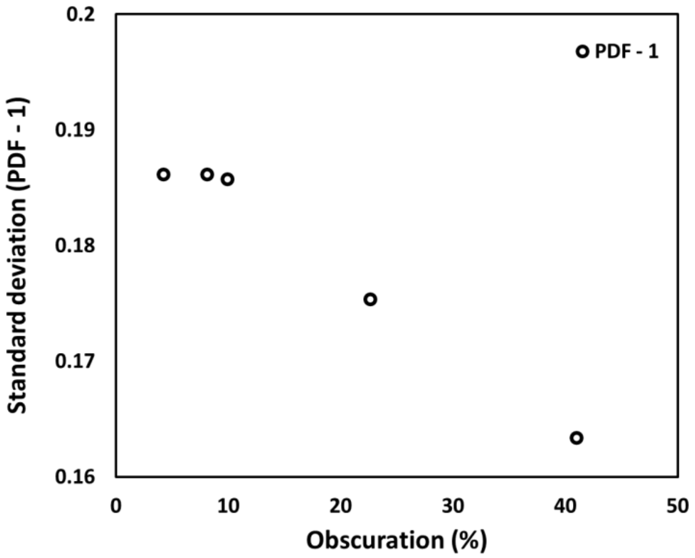

Figure S5: Standard deviation of larger droplets plotted against laser obscuration (%).

Section S3: Correlations for estimating pressure drop[1]

The physical model to estimate the viscosity of emulsion is related to the pressure drop (in terms of Euler number, Eu) and Reynold number (Re) based on Thaker et al.[1] work for range of viscosity and device scale three different flow regimes was observed with Eu and Re.

Laminar regime: $Eu^* = \frac{a}{Re^*}$ if $Re^* < Re^*_{12}$ (S-2)

Transient regime: $Eu^* = b\,(Re^*)^c$ if $Re^*_{12} \leq Re^* < Re^*_{23}$ (S-3)

Turbulent regime: $Eu^* = d$ if $Re^* \geq Re^*_{23}$ (S-4)

The values of the Euler number and Reynolds number are scaled using the following:

$$Eu^* = \frac{Eu}{50} \quad \text{(S-5)}$$

$$Re^* = \frac{Re}{1000} \quad \text{(S-6)}$$

In equation S-2, S-3 and S-4, Re^*_{12} and Re^*_{23} are boundaries between the laminar and transient, and turbulent regimes respectively. Equations for boundaries between regimes:

$$Re^*_{12} = \left(\frac{a}{b}\right)^{1/(1+c)} \quad \text{(S-7)}$$

$$Re^*_{23} = \left(\frac{d}{b}\right)^{1/c} \quad \text{(S-8)}$$

In the laminar regime parameter, a, is not a constant but is a function of scale and viscosity. The data indicated following relationship for a.

$$a = \frac{5d^*_T}{\mu^*} \quad \text{(S-9)}$$

$$b = 0.35, c = 0.4\ and\ d = 1$$

Where,

$$d^*_T = \frac{d_T}{6} \quad \text{(S-10)}$$

$$\mu^* = \frac{\mu}{\mu_w} \quad \text{(S-11)}$$

Section S4: Correlations between turbidity and absorbance

The relationship between turbidity (in NTU), and absorbance (*A*) for the same oil volume fractions is illustrated in Figure S6, where the absorbance (in m^{-1}) is approximately 532 times less than the observed turbidity (in NTU).

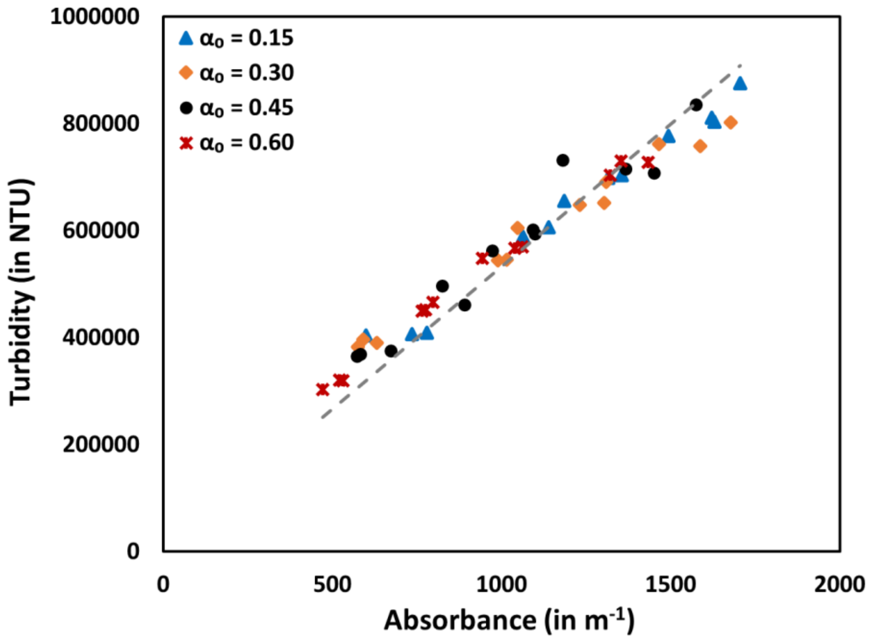

Figure S6. Correlation between turbidity (in NTU) versus absorbance (*A*) relationship for emulsions with different oil volume fractions and number of passes.

Section S5: Correlations between the Sauter mean diameter and the effective diameter

The measured Sauter mean diameter (d_{32}) using laser diffraction technique and effective characteristic diameter, d_{eff} estimated from turbidity measurements are found to be linearly related as shown in Figure S7.

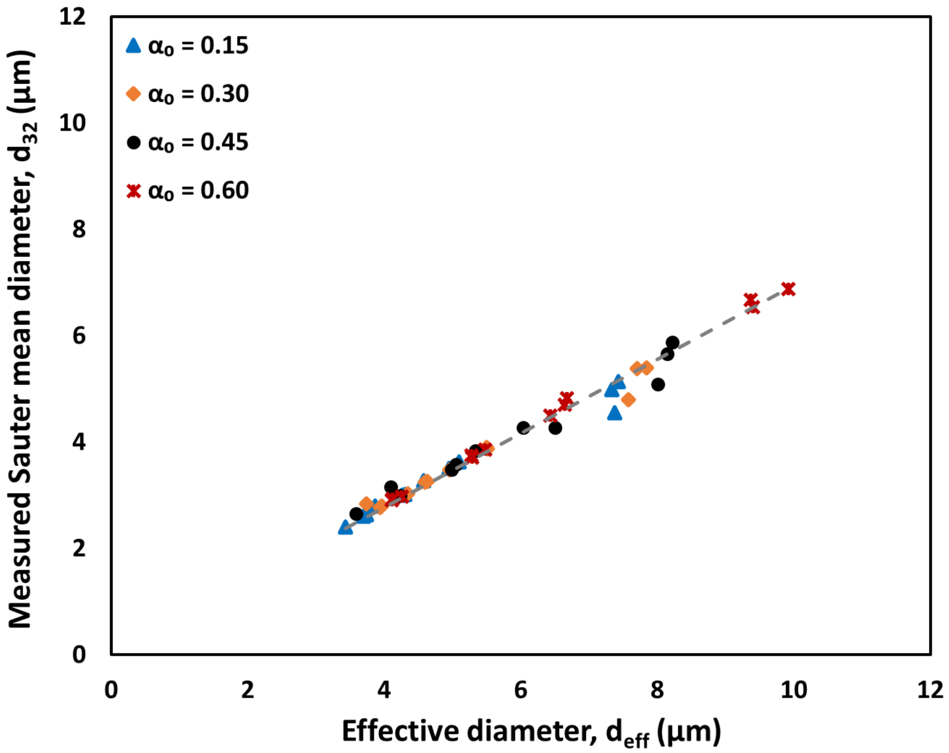

Figure S7. Measured Sauter mean diameter (d_{32}) versus effective diameter (d_{eff}) estimated from turbidity (in NTU) measurements for emulsions with different oil volume fractions and number of passes.

Table S1. Three log-normal fitting parameters for different number of passes and characteristic droplet diameters (in micron).

α_o	n	W_1	W_2	μ_1	σ_1	μ_2	σ_2	μ_3	σ_3	d_{21}	d_{32}	d_{10}	d_{20}	d_{30}	d_{40}	d_{50}	d_{60}	d_{70}	d_{80}	d_{90}
**0.15**	1	0.03	0.17	-0.22	0.23	0.61	0.49	2.9	0.75	1.5	4.9	1.6	3.6	7.4	10.7	13.9	17.2	21.1	26.7	36.3
	5	0.05	0.28	-0.22	0.23	0.68	0.52	2.4	0.51	1.4	3.5	1.3	2.1	3.6	6.0	8.0	9.9	11.8	14.1	17.3
	20	0.05	0.33	-0.23	0.23	0.66	0.50	2.2	0.45	1.4	2.9	1.2	1.8	2.7	4.2	5.8	7.3	8.7	10.3	12.7
	100	0.06	0.31	-0.24	0.23	0.53	0.46	1.9	0.47	1.4	2.5	1.1	1.6	2.3	3.2	4.4	5.4	6.6	7.8	9.6
**0.3**	1	0.03	0.15	-0.22	0.22	0.58	0.48	2.8	0.74	1.5	5.2	1.7	4.2	7.9	11.0	14.1	17.2	21.0	26.0	34.5
	5	0.04	0.25	-0.22	0.23	0.66	0.51	2.4	0.52	1.5	3.8	1.4	2.4	4.3	6.7	8.6	10.4	12.4	14.8	18.0
	20	0.04	0.31	-0.24	0.22	0.66	0.50	2.2	0.46	1.4	3.2	1.2	2.0	3.1	4.9	6.5	7.9	9.4	11.1	13.5
	100	0.05	0.32	-0.24	0.23	0.62	0.48	2.0	0.46	1.4	2.8	1.1	1.8	2.6	3.8	5.1	6.3	7.6	9.0	11.1
**0.45**	1	0.03	0.12	-0.26	0.21	0.51	0.46	2.8	0.73	1.5	5.5	1.9	5.4	8.6	11.4	14.3	17.3	21.3	26.7	36.6
	5	0.03	0.19	-0.26	0.22	0.57	0.49	2.4	0.52	1.5	4.1	1.5	3.1	5.8	7.7	9.4	11.1	13.0	15.4	18.8
	20	0.04	0.23	-0.28	0.21	0.56	0.48	2.2	0.45	1.4	3.4	1.3	2.2	4.1	5.8	7.1	8.4	9.8	11.4	13.8
	100	0.05	0.25	-0.28	0.22	0.49	0.45	1.9	0.46	1.4	2.9	1.1	1.9	3.0	4.2	5.3	6.3	7.3	8.6	10.4
**0.6**	1	0.10	0.09	-0.25	0.21	0.48	0.44	2.9	0.66	1.6	6.7	2.6	7.8	10.7	13.4	16.2	19.6	24.0	31.0	47.1
	5	0.14	0.14	-0.27	0.22	0.51	0.47	2.5	0.48	1.5	4.7	1.7	4.8	7.2	8.8	10.4	11.9	13.7	16.0	19.3
	20	0.17	0.17	-0.28	0.21	0.47	0.45	2.2	0.43	1.5	3.8	1.4	3.1	5.3	6.5	7.7	8.8	10.0	11.6	13.7
	100	0.19	0.20	-0.30	0.20	0.34	0.40	1.8	0.42	1.4	2.9	1.1	2.0	3.5	4.5	5.3	6.1	7.0	8.1	9.6

Note: In Sauter mean diameter (d_{32}) associated average standard deviation across all the passes [±0.15 - oil volume fraction, $\boldsymbol{\alpha_o}$- 0.15]; [±0.15 - oil volume fraction, $\boldsymbol{\alpha_o} - 0.30$;[±0.22 - oil volume fraction, $\boldsymbol{\alpha_o} - 0.45$]; [±0.09 - oil volume fraction, $\boldsymbol{\alpha_o}$ - 0.60].

Notations:

d_T	throat diameter, mm
d_T^*	dimension less throat diameter (-)
d_{32}	Sauter mean diameter (μm)
d_{eff}	droplet effective diameter (m)
μ	viscosity (kg/ms)
μ_w	water viscosity (kg/ms)
μ^*	dimension less viscosity (-)
b	parameter constant (-)
c	parameter constant (-)
d	parameter constant (-)
d_x	diameter corresponding to characteristic droplet diameters (μm)
α_o	Oil volume fraction, (-)
n	Number of pass (-)
ε_o	oil volume fraction in the turbidity vial (-)

Acronyms

DSD	droplet size distribution
HC	hydrodynamic cavitation
NTU	nephelometric turbidity units
PDF	probability density function

References:

1. Thaker, A. H.; Madane, K. R.; Ranade, V. V. Influence of viscosity and device scale on pressure drop and cavitation inception: Vortex based cavitation devices. *Chemical Engineering Journal* **2023**, 474, 145943.
